# DE-MRI allows comparison of lesion formation after pulmonary vein isolation with different ablation catheters in patients with paroxysmal atrial fibrillation

**DOI:** 10.1186/1532-429X-15-S1-O90

**Published:** 2013-01-30

**Authors:** Christian Mahnkopf, Nathan S Burgon, Philipp Halbfass, Oliver Turschner, Anya G Mihaylova, Eugene Kholmovski, Johannes Brachmann, Nassir F Marrouche

**Affiliations:** 1 Dept. of Cardiology, Klinikum Coburg, Coburg, Germany; 2 CARMA-Center University of Utah, Salt Lake City, UT, USA

## Background

We compared the difference in left atrial tissue remodeling (LATR) pre-ablation and post-ablation lesion characteristics between three methods for electrical isolation of pulmonary veins [cryoballoon (cryo), pulmonary vein ablation catheters (PVAC) and single-tip radiofrequency (SRF)] routinely done to treat paroxysmal atrial fibrillation (PAF).

## Methods

Patients presenting with PAF who qualified for a cryo, PVAC or SRF ablation were prospectively followed. DE-MRI of the left atrium (LA) was performed prior to and three months post procedure. The degree of LATR is reported as a percentage of the total LA area.

## Results

**Figure 1:**
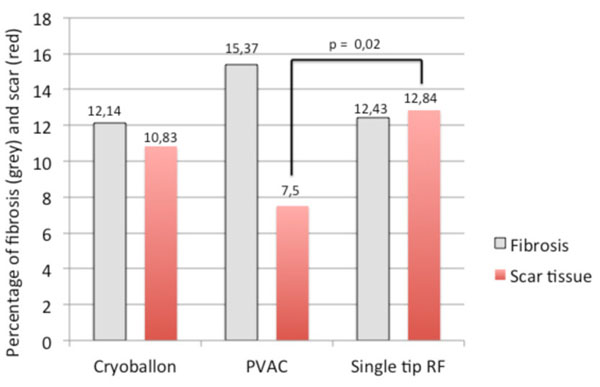
Percentage of pre-ablation LA fibrosis (grey) and amount of scar tissue (red)

**Figure 2:**
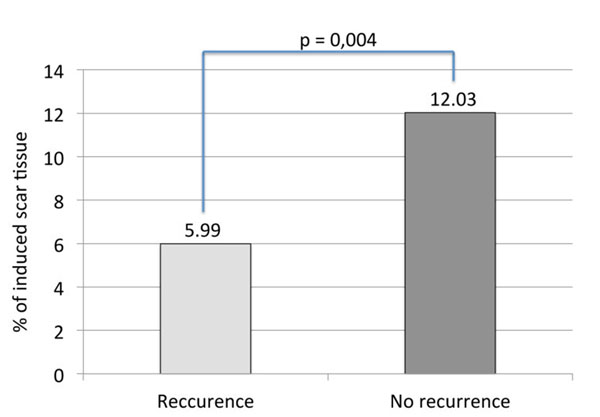
Percentage of scar tissue in patients with and without reccurence

**Figure 3:**
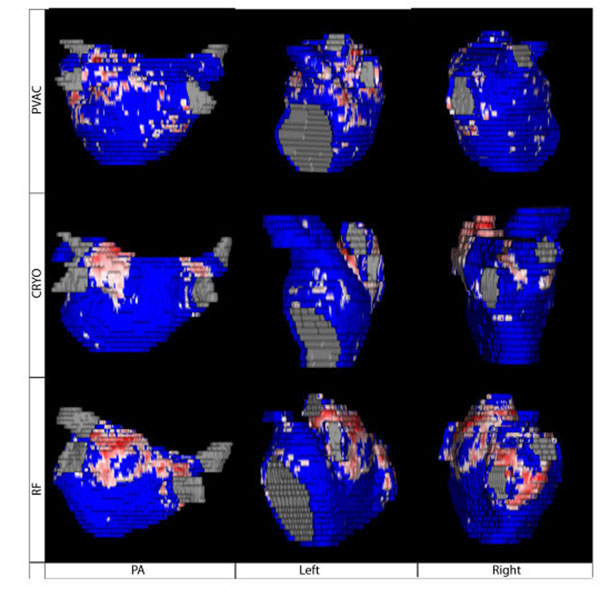
Lesion formation after PVAC (top), Cryo- (middle) and RF-ablation (lower row). Blue areas are healthy myocardium. Red areas reflect scar tissue.

37 patients (26 males, mean age = 63±10.12 years) were included in this study. Six patients underwent an ablation using PVAC catheter, SRF catheter was used in 14 patients, and 17 patients underwent a cryoballoon ablation. Pre-ablation LATR was comparable in all three cohorts (Figure 1). Extent of scar tissue was higher in Cryo and SRF patients compared to PVAC patients (Figure1). Overall six patients were found to have AF recurrence at 3-months follow-up. Patients with recurrence had a significantly lower amount of ablation lesions than patients in sinus rhythm (5.93% vs. 15.98%; P=0,004; Figure 2). 3-dimensional visualization allows comparison of induced lesions by different ablation catheters (Figure 3).

## Conclusions

From our preliminary results, PVAC ablation appears to result in lesser scar formation as compared to Cryo and SRF ablation. The greater recurrence in patients with low scar post-ablation suggests the need to implement an adequate ablation strategy that results in greater scar to maximize successful outcomes. DE-MRI is an appropriate method to compare lesion formation induced by different ablations strategies.

## Funding

None.

